# Biomaterials for orbital fractures repair

**Published:** 2015

**Authors:** M Totir, R Ciuluvica, I Dinu, I Careba, S Gradinaru

**Affiliations:** * University Emergency Hospital Bucharest, Bucharest, Romania; ** Anatomy Department, “Carol Davila” University of Medicine and Pharmacy, Bucharest, Romania; *** Ophthalmology Department, “Carol Davila” University of Medicine and Pharmacy, Bucharest, Romania

**Keywords:** orbital fracture, titanium mesh, bone graft, reconstruction

## Abstract

The unique and complex anatomy of the orbit requires significant contouring of the implants to restore the proper anatomy. Fractures of the orbital region have an incidence of 10-25% from the total facial fractures and the most common age group was the third decade of life.

The majority of cases required reconstruction of the orbital floor to support the globe position and restore the shape of the orbit. The reason for this was that the bony walls were comminuted and/ or bone fragments were missing. Therefore, the reconstruction of the missing bone was important rather than reducing the bone fragments. This could be accomplished by using various materials. There is hardly any anatomic region in the human body that is so controversial in terms of appropriate material used for fracture repair: non resorbable versus resorbable, autogenous/ allogeneic/ xenogenous versus alloplastic material, non-prebent versus preformed (anatomical) plates, standard versus custom-made plates, nonporous versus porous material, non-coated versus coated plates. Thus, the importance of the material used for reconstruction becomes more challenging for the ophthalmologist and the oral and maxillofacial surgeon.

## Introduction

Fractures of the orbital region have an incidence of 10-25% from the total facial fractures [**1**] and the most common age group was the third decade of life (29%) [**2**]. The most common etiology seems to be violent assault or nonviolent traumatic injury (49.4%) [**2**] and the most frequent fracture involved, the zygoma (23.6%), followed by the orbital floor (21.4%), maxilla, mandible and nasal bones [**3**]. For these patients, modern imaging analysis offers a unique chance to quantitatively asses the surgical result and stability over the time. This can provide valuable information for future recommendation [**4**]. The careful assessment of the defect size should be performed preoperatively with the CT scan in the sagittal view, which is in the course of the orbital nerve, plus the coronal view [**5**].

Jaquiéry differentiated between the following classes in orbital trauma [**6**]:

Class I: Small, isolated defects of the orbital floor or the medial orbital wall of approx. 1 – 2 cm2.

Class II: Defects of the orbital floor and/ or the medial orbital wall > 2 cm2, bony structures of the medial wall of the infraorbital fissure are intact.

Class III: Defects of the orbital floor and/ or the medial orbital wall > 2 cm2, without bony structures of the infraorbital fissure.

Class IV: Defects of the whole orbital floor and the medial wall to the infraorbital fissure.

The timing of surgery has also been debated over the years. Except for the circumstance of a trapdoor fracture with the potential of an ischemic contracture of the entrapped tissue, generally, several days are allowed for orbital and eyelid edema to resolve. This delay also allows a more accurate assessment of extraocular muscle function [**7**].

As demonstrated by studies, there is a lack of consensus in recognizing one material as the optimal one for orbital reconstruction. The available products are the following:

1. Titanium meshes present a series of advantages [**8**,**9**]. In 2009, Scollozzi revealed in his paper, a high rate of success with an acceptable rate of major clinical complications (10%) and an anatomic restoration of the bony orbital contour and volume that closely approximated that of the contralateral uninjured orbit [**10**].

**Advantages:**

• Availability

• Stability

• Contouring (eased by the artificial sterile skull)

• Adequate in large three-wall fractures (the pre-bent plate is limited to medial wall and orbital wall fractures only).

• Radio-opacity

• Spaces within the mesh to allow dissipation of fluids

• No donor site needed

• Tissue incorporation may occur

**Disadvantages:**

• Costs

• Possible sharp edges if not properly trimmed

2. Bone graft: in 2012, a paper by Zunz et al concluded that the construction of the orbital floor fractures after trauma by using autologous bone grafts is safe and associated with a low rate of complications [**11**].

**Advantages:**

• Low material costs

• Smooth surface

• Variability in thickness

• Radio-opacity

• Maximal biocompatibility

• Periorbita readily dissects off the bone in secondary reconstructions

**Disadvantages:**

• Additional donor site needed (necessitating additional surgery time for harvest, pain, scar and possible surgical complications)

• Possible contour and dimensional changes due to remodeling

• Difficult to shape according to patients’ anatomy

• Less drainage from the orbit than with titanium mesh

3. Porous polyethylene sheets (PPE). In a study published by Lin, it was demonstrated that porous polyethylene implants in the repair of orbital wall fractures had good results with few complications [**12**].

**Advantages:**

• Availability

• Contouring (eased by the artificial sterile skull)

• Smooth edges

• Allows tissue ingrowth

**Disadvantages:**

• Not radiopaque (not visible on postoperative images)

• Lack of rigidity when a very thin wafer of PPE is used. When a thicker rigid wafer is used, there is a risk of causing a dystopia.

• Less drainage from the orbit than with a titanium mesh

4. Composite of porous polyethylene and titanium mesh

By combining titanium mesh with porous polyethylene, the material becomes radiopaque and more rigid than the porous polyethylene of a similar thickness. Some surgeons also believe that there is less risk of having retained sharp barbs, which can lead to an entrapment of soft tissues during placement [**13**].

**Advantages:**

• Availability

• Stability

• Contouring (eased by the artificial sterile skull)

• Adequate in large three-wall fractures (the pre-bent plate is limited to medial wall and orbital wall fractures only).

• Radio-opacity

• No donor site needed

• Tissue incorporation may occur

**Disadvantages:**

• Less drainage from the orbit than with titanium mesh

5. Resorbable materials: Thermoplastic and non-thermoplastic materials

Thermoplastic blends of cornstarch material with ethylene vinyl alcohol copolymers reinforced with hydroxyapatite were used based on their mechanical properties and their modulus closed to that of human bone [**14**].

**Advantages:**

• Availability

• **Handling/ contourability (only for thermoplastics)**

• **Smooth surface** and smooth edges

**Disadvantages:**

• No radio-opacity

• Degradation of material with possible contour loss

• Sterile infection/ inflammatory response

• Difficult to shape according to patients’ anatomy (only for non-thermoplastics)

• Less drainage from the orbit than with uncovered titanium mesh (in case when non-perforated material is used)

6. Preformed orbital implant: Bittermann showed that by using computer-assisted techniques, anatomically preformed orbital implants and intraoperative imaging, the surgeon could have precise and predictable results of orbital reconstructions [**15**].

**Advantages:**

• Radio-opacity

• Smooth surface

• Minimal or no contouring necessary

**Disadvantages:**

• Cost

The first step in a choice for an implant is to focus on its most important features in order to reduce complications incidence.

These features should be lightweight, porosity (the implant must allow vascular orbital tissues to invade its structure), biocompatibility (the implant has to be tolerated and accepted by the orbital tissues), low rate complications, easy to insert, economic cost. **Table 1** illustrates factors influencing the decision for the implant choice.

**Table 1:** Factors influencing decision for the choice of the material

Surgeon experience
Severity of fracture
Individual characteristics
Cost

The multitude materials with different results in published studies showed that we do not have the answer for the best type of implant for orbital reconstruction. With the increasing need to develop clean, non-toxic and environmentally friendly techniques, hydroxyapatite powders have been extracted by using bioproducts from marine sources (e.g. coral, cuttlefish shells), animal teeth and bones (porcine, bovine), natural gypsum or natural calcite [**16**,**17**]. Compared with hydroxyapatite produced by synthetic methods, hydroxyapatite partially or entirely generated from biogenic sources is supposed to be accepted better by the living organisms, because of its physic-chemical similarity to the human bone apatite. 

**Acknowledgement**

This paper was supported by the Sectoral Operational Programme Human Resources Development (SOP HRD), financed from the European Social Fund and by the Romanian Government under the contract number POSDRU/159/1.5/S/132395”.
